# Feasibility and safety of continuous retrograde administration of Del Nido cardioplegia: a case series

**DOI:** 10.1186/s13019-015-0383-x

**Published:** 2015-11-26

**Authors:** Marc Najjar, Isaac George, Hirokazu Akashi, Takashi Nishimura, Halit Yerebakan, Linda Mongero, James Beck, Stephen C. Hill, Hiroo Takayama, Mathew R. Williams

**Affiliations:** Department of Surgery, Division of Cardiothoracic Surgery, College of Physicians and Surgeons of Columbia University, New York Presbyterian Hospital, MHB 7GN-435, 177 Fort Washington Ave, New York, 10032 NY USA

**Keywords:** Myocardial protection, Cardioplegia, Reoperation, Outcomes, Patient safety

## Abstract

**Background:**

Del Nido (DN) cardioplegia, a calcium-free, hyperkalemic solution containing lidocaine and magnesium has been developed to help reduce intracellular calcium influx and the resulting myocyte damage in the immediate postischemic period following cardiac arrest. DN cardioplegia has been used for pediatric cardiac surgery but its use in complex reoperative surgery has not been studied. We specifically report the outcomes of patients undergoing reoperative cardiac surgery after previous coronary artery bypass grafting with a patent internal mammary artery (IMA).

**Methods:**

Patients undergoing reoperative cardiac surgery with prior coronary bypass grafting surgery were studied between 2010 and 2013. Fourteen patients were identified who required continued retrograde cardioplegia administration. In all cases, an initial antegrade dose was given, followed by continuous retrograde administration. Demographics, co-morbidities, intra-operative variables including cardioplegia volumes, post-operative complications, and patient outcomes were collected.

**Results:**

The mean age of all patients was 73.3+/−6.7 years, and 93 % were male. Aortic cross clamp time and cardiopulmonary bypass times were 81+/−35 and 151+/−79 mins, respectively. Antegrade, retrograde and total cardioplegia doses were 1101+/−398, 3096+/−3185 and 4367+/−3751 ml, respectively. An average of 0.93+/−0.92 inotropes and 1.50+/−0.76 pressors were used on ICU admission after surgery. ICU and total hospital lengths of stay were 5.5+/−7.4 and 9.6+/−8.0 days, respectively. Complications occurred in two patients (14 %) (pneumonia and prolonged mechanical ventilation) and new arrhythmias occurred in five patients (36 %) (four new-onset atrial fibrillation and one pulseless electrical activity requiring 2 min of chest compression). No perioperative myocardial infarctions were noted based on electrocardiograms and cardiac serum markers. Postoperatively, left ventricular function was preserved in all patients whereas two patients (14 %) had mild decrease in right ventricular function as assessed by echocardiography. No mortality was observed.

**Conclusion:**

Del Nido cardioplegia solution provides acceptable myocardial protection for cardiac surgery that requires continuous retrograde cardioplegia administration. DN cardioplegia’s administration in a continuous retrograde fashion with a patent IMA is believed to provide adequate myocardial protection while avoiding injuring the IMA through dissection and clamping.

## Background

Retrograde cardioplegia via the coronary sinus has become an essential method to deliver cardioplegia to the left ventricle, particularly in the setting of critical coronary stenosis, aortic insufficiency, or other factors precluding antegrade cardioplegia administration [1]. Patent bypass grafts after coronary artery bypass grafting (CABG), such as an internal mammary artery (IMA) graft, can prevent full cardioplegic arrest if unable to be isolated and clamped; in this situation, continuous retrograde cardioplegia has been administered using whole blood cardioplegia with reduced potassium concentration to achieve ventricular fibrillatory arrest. The most popular technique to deal with a patent IMA is to clamp both the aorta and the patent graft and administer antegrade and retrograde cardioplegia [2]. However, if an area of the myocardium is very dependent on the patent IMA, clamping the graft may cause inadequate myocardial protection. Moreover, the dissection and clamping of the IMA have been linked with the associated morbidity and mortality of the procedure as they could cause inadvertent injury to the graft [3]. Other groups have reported satisfactory results with selective use of continuous retrograde cardioplegia administration in patients whose IMA was deemed technically challenging to safely dissect and clamp [2, 4].

Del Nido cardioplegia (DN) (Compass- Baxter Healthcare Inc., Edison, NJ) has been in use for over 20 years in the pediatric populations. It is delivered cold (4 °C) at a ratio of one part blood to four parts crystalloid, and achieves arrest using a low calcium, hyperkalemic solution with lidocaine and magnesium additive for a total volume of 1 L [5–8]. After mixture of the blood and crystalloid components, the final concentrations of Na^+^, K^+^ and Ca^2+^ in the DN solution are respectively: 38, 22 and 0.24 mmol/L. The safety of its use in adult cardiac surgery has been recently reported in three series from our institution evaluating specific situations of cardiac surgeries [9–11]. These studies showed that DN cardioplegia could be safely used in patients undergoing CABG for acute myocardial infarction (AMI), aortic valve replacement (AVR) or isolated reoperative AVR. They all showed that morbidity and mortality in patients receiving DN cardioplegia is comparable to those of patients receiving standard whole blodd (WB) cardioplegia. We report our use of DN cardioplegia given as a continuous retrograde infusion in the setting of complex, reoperative cardiac surgeries with a patent prior IMA unable to be isolated. DN solution given in a continuous retrograde fashion is, unlike the original solution, made of four units of blood to one unit of crystalloid in order to reduce hemodilution.

## Methods

All patients who underwent reoperative cardiac surgeries after prior CABG between 2010 and 2013 were reviewed retrospectively, and a total of 14 patients were identified who received continuous retrograde DN. In all cases, a patent IMA was present and was not clamped during cross-clamp. Patient characteristics were collected, including age, gender, body surface area, pre-operative co-morbidities and surgical risk factors [hyperlipidemia, prior myocardial infarct (MI), hypertension, stroke, chronic obstructive pulmonary disease, chronic kidney disease with baseline creatinine >2.0 mg/dL, atrial fibrillation, congestive heart failure by New York Heart Association functional classification, prior CABG], left ventricular ejection fraction (LVEF), number of inotropes and vasopressors on intensive care unit (ICU) admission, days on ventilator, length of postoperative ICU stay (ICULOS), total post-operative hospital length of stay (THLOS), in-hospital mortality, and post-operative complications [transfusion, sepsis or endocarditis, sternal wound infection, gastrointestinal bleeding, MI, reoperation for bleeding, acute renal injury, stroke, transient ischemic attach, need for permanent pacemaker, prolonged mechanical ventilation and need for reintubation]. This study met all guidelines of the Institutional Review Board of Columbia University Medical Center.

Using mild systemic hypothermia (32–34 ° C), antegrade cardioplegia was first administered after cross clamp (XC) (500–1000 cc), followed by 500–750 cc retrograde. DN cardioplegia was the only cardioplegia solution used and was delivered at a temperature of 4 °C. For the initial antegrade and retrograde doses, the solution was administered as a 1:4 blood to crystalloid ratio with concentrations of Na^+^, K^+^, Ca^2+,^ Mg^2+^ and lidocaine of respectively: 38, 22, 0.24, 6.6 and 104 mg/L. Once ventricular fibrillation was achieved (typically at approximately 1 L), cardioplegia was switched to continuous retrograde using a 4:1 blood to crystalloid ratio to prevent hemodilution, the modified solution has concentrations of Na^+^, K^+^, Ca^2+,^ Mg^2+^ and lidocaine of respectively: 115, 9.2, 1, 2.4 and 26 mg/L (Tables 1 and 2). Retrograde flow was maintained at approximately 40 ml/kg/min for a coronary sinus pressure of 20–30 mmHg. Particular attention was made to continuously check the coronary sinus balloon pressure and maintain it at 20–30 mmHg and to frequently watch the left main coronary ostia to check for blood return. No redosing was required. However, if significant electrical activity was encountered, a full 1:4 dose cardioplegia dose would have been given retrograde or via the coronary ostia.

**Table 1:** Composition of del Nido Cardioplegia (before mixing with blood)

	Whole Blood Cardioplegia	del Nido Cardioplegia
Carrier (1 L)	D5W	Plasma-lyte A
Blood: Crystalloid ratio	4:1	1:4
KCl (mEq)	80	26
NaHCO_3_ (mEq)	30	13
Mannitol (g)	12.5	3.3
Lidocaine (mg)	0	130
Magnesium (g)	0	2

**Table 2:** Final concentrations in del Nido Cardioplegia solutions (after mixing with blood)

	Initial single dose antegrade and retrograde	Continuous retrograde
Carrier	Plasma-lyte A	Plasma-lyte A
Blood: Crystalloid ratio	1:4	4:1
K^+^ (mmol/L)	22	9.2
Na^+^ (mmol/L)	38	115
Ca^2+^ (mmol/L)	0.24	1.0
Lidocaine (mg/L)	104	26
Mg^2+^ (mmol/L)	6.6	2.4

Cardioplegia was intermittently interrupted for less than 5 min for the purposes of visualization, especially during valve debridement and when placing annular sutures in the left coronary sinus. The operative details that were collected included cardiopulmonary bypass (CPB) and XC time, ischemic time, doses of antegrade and retrograde cardioplegia, lowest body temperature and number of transfusions.

## Results

### Patient demographics

The baseline pre-operative patients’ characteristics are summarized in Table 3. Mean age was 73.3 ± 6.7 years (range 61–83 years). Thirteen patients (93 %) were males, and most of them (86 %) had hypertension and dyslipidemia. Mean LVEF was 44 ± 16 %. The types of reoperative surgeries were as follows: nine isolated Aortic Valve Replacement (AVR), 1 isolated Mitral Valve Replacement (MVR), 1 AVR/MVR/Tricuspid Valve Repair (TVr), 1 CABG/AVR/Mitral Valve repair (MVr), 1 MVR/TVr, and 1 Aortic dissection repair. Mean Society of Thoracic Surgeon predicted risk of mortality score was 4.3 ± 2.1.

**Table 3:** Baseline Demographics

Number	14
Age, year (mean ± SD)	73.3 ± 6.7
Male, n (%)	13 (93)
BMI, kg/m^2^ (mean ± SD)	27 ± 4.0
BSA m^2^ (mean ± SD)	1.97 ± 0.16
Diabetes, n (%)	5 (36)
Hypertension, n (%)	12 (86)
Dyslipidemia (%)	12 (86)
COPD, n (%)	2 (14)
PVD, n (%)	4 (29)
Stroke, n (%)	2 (14)
Atrial fibrillation, n (%)	5 (36)
Creatinine, > 2 (mg/dL) n (%)	2 (14)
LVEF, % (mean ± SD)	44 ± 16
NYHA grade (mean ± SD)	2.1 ± 1.1
STS score (mean ± SD)	4.3 ± 2.1

Definitions: *BMI* body mass index, *BSA* body surface area, *COPD* chronic obstructive pulmonary disease, *PVD* peripheral vascular disease, *LVEF* left ventricular ejection fraction, *NYHA* New York Heart Association, *STS* society of thoracic surgery

### Intra-operative variables

Total CPB and XC times were within an acceptable range, 151 ± 79 and 81 ± 35 min respectively, and no intraoperative complications related to cardioplegia administration were noted. Antegrade, retrograde and total cardioplegia doses were 1101 ± 398, 3096 ± 3185 and 4367 ± 3751 ml, respectively. Ventricular fibrillation was achieved in all patients. Mechanical circulatory support was not required in any patient whereas an intra-aortic balloon pump was placed in only one patient. The intraoperative measurements are summarized in Table 4.

**Table 4:** Intraoperative measurements

Cross-clamp time (min, mean ± SD)	81 ± 35
CPB time (min, mean ± SD)	151 ± 79
ACP dose (mL, mean ± SD)	1101 ± 398
RCP dose (mL, mean ± SD)	3096 ± 3185
TCP dose (mL, mean ± SD)	4367 ± 3751
Lowest temperature (°C, mean ± SD)	32.36 ± 3.67

Definitions: *CPB* cardiopulmonary bypass, *ACP* antegrade cardioplegia, *RCP* retrograde cardioplegia, *TCP* total cardioplegia

### Postoperative outcomes

Postoperative complications occurred in two patients (14 %), both had pneumonia complicated by sepsis and a prolonged mechanical ventilation time (8 and 24 days). Five patients (36 %) had new arrhythmias postoperatively (four had new-onset atrial fibrillation and one had pulseless electrical activity requiring 2 min of chest compression). ICULOS and THLOS were respectively 5.5 ± 7.4 and 9.6 ± 8.0 days. On average, patients needed 0.93 ± 0.92 inotropes and 1.50 ± 0.76 vasopressors on ICU admission. No perioperative MIs (PMI) [12] were diagnosed. Biomarkers were ordered whenever symptoms and/or EKG signs of possible PMI were suspected, as well as when patients were enrolled in clinical trials mandating serial cardiac markers level assessment. Troponin I and CKMB were ordered for four patients (29 %), of which actually only one had a suspicion of PMI, the other three were part of clinical trials. Average postoperative Troponin I was 1.01 ± 0.335 ng/mL (reference range 0.00–0.008 ng/mL) and CKMB 3.58 ± 3.47 ng/mL (reference range 0.5–5.5 ng/mL). Immediate preoperative and postoperative trans-esophageal echocardiographs (TEE) as well as predischarge transthoracic echocardiographs were reviewed in all patients to assess ventricular function preservation. All patients had preservation of left ventricular (LV) function and only three patients (21 %) had slightly worsened right ventricular (RV) function immediately postop (no dysfunction preop to mild dysfunction postop), which in 1 case (7 %) recovered completely by the time the patient was discharged. Moreover, two patients (14 %) had an improvement in RV function from preop to postop with a mild dysfunction not appreciated on the postop TEE. All patients were discharged from the hospital alive for a 0 % 30-day and in-hospital mortality. Follow-up duration was 361 ± 356 days and midterm results were excellent with a 100 % survival rate. Moreover, on follow-up echocardiography, there was preservation of LV function in all 14 patients and no new RV dysfunction. Postoperative variables are summarized in Table 5.

**Table 5:** Postoperative variables

Inotropes on ICU admission, number (mean ± SD)	0.93 ± 0.92
Pressors on ICU admission, number (mean ± SD)	1.50 ± 0.76
Ventilation, days (mean ± SD)	3.6 ± 6.5
PRBCs, units (mean ± SD)	2.86 ± 4.96
Post-op EF, % (mean ± SD)	45 ± 14
ICULOS, days (mean ± SD)	5.5 ± 7.4
THLOS, days (mean ± SD)	9.6 ± 8.0
Complications, n (%)	2 (14)
New arrhythmia, n (%)	5 (36)
30-days Mortality, n (%)	0 (0)
Follow-up duration, days (mean ± SD)	361 ± 356
Cumulative mortality, n (%)	0 (0)

Definitions: *PRBCs* packed red blood cells, *EF* ejection fraction, *ICULOS* intensive care unit length of stay, *THLOS* total hospital length of stay

## Discussion

The patent IMA graft poses a special concern for surgeons - its dissection and isolation can pose risk, and injury to this vessel during surgery is associated with extremely high rates of mortality [13]. Thus, continuous retrograde administration of cardioplegia in the midst of a patent IMA has become an accepted technique for effective myocardial preservation [14]. Other strategies to manage myocardial protection during reoperative cardiac surgeries with a patient IMA have been described. The most popular technique is clamping both the aorta and the patent IMA graft with antegrade and retrograde infusion of cardioplegia. The main limitation of that technique are the fact that some areas of the myocardium might be dependent on the patent graft and simply clamping it may lead to suboptimal myocardial protection, as well as the risk of injury to the graft that comes with dissection and clamping [2, 3]. Other strategies that have been described but have not gained widespread popularity are: deep hypothermia alone [15], continuously perfused beating heart [16], endovascular occlusion or supraclavicular clamping of the patent IMA [17, 18]. Despite providing adequate control of blood flow through the graft, manipulation of the graft by itself can cause injury and should probably be avoided whenever possible. Here we report our experience with the combination of antegrade, retrograde and continuous retrograde hypothermic cardioplegia through a patent IMA using Del Nido cardioplegia, a single dose hyperkalemic agent. Our data suggests that we achieved effective myocardial preservation using continuous retrograde administration, as evidenced by the absence of perioperative myocardial infarction, a good LV and RV function preservation, reasonable CPB and XC times, low amount of vasoactive medications post-bypass, and the lack of need for additional mechanical circulatory assistance. Thus, the strategy of antegrade, retrograde, and continuous retrograde cardioplegia administration provided sufficient protection for surgery to be completed without significant hindrance. Spontaneous recovery of rhythm occurred in all patients, and there was only one case of low cardiac output syndrome which required the intraoperative insertion of an IABP. Part of these positive outcomes may be attributed to the fact that the anterior wall of the LV is continuously perfused via the IMA. Nonetheless, use of DN cardioplegia does offer some distinct advantages, such as uninterrupted conduct of surgery, stabilization of calcium handling regulation during the ischemic period [19], less need for systemic cooling and a lower total volume of cardioplegia.

Although not fully understood yet, ischemia-reperfusion injury is thought to be at least partly due to an increase in intracellular calcium concentrations in postischemic myocytes. This increase in Ca^2+^ is related to an increase in intracellular Na^+^ during the ischemic period, causing the Na^+^/ Ca^2+^ channel to increase calcium influx [20-22] . As a result of high levels of intracellular Ca^2+^, the myocytes hypercontract causing irreversible damage to the cytoskeleton and even cell death [20] . Therefore, Del Nido cardioplegia provides a theoretical advantage over other cardioplegia solutions in that it is calcium-free and contains lidocaine, an Na^+^ channel blocker, as well as magnesium, a calcium competitor. All of these components contribute to limiting calcium influx in the postperfusion phase. The effectiveness of these mechanisms has been demonstrated in both animal and pediatric hearts, while DN-arrested rats hearts had a significantly lower intracellular calcium levels when compared to hearts arrested with regular whole blood (WB) cardioplegia [23], pediatric patients receiving DN solution had in turn a reduction in troponin T release following surgery compared to patients receiving the WB solution [19].

A lower total volume of cardioplegia allows a reduction of hemodilution while on bypass and consequently a decreased need for transfusion. The advantages of decreasing hemodilution have been demonstrated with WB or undiluted blood (microplegia) cardioplegia solutions, which offer a better myocardial protection [24] and limit myocardial edema [25] compared with crystalloid cardioplegia.

## Conclusion

As a conclusion, del Nido cardioplegia solution can be used as a safe alternative to whole blood solutions in cardiac surgeries requiring the administration of continuous retrograde cardioplegia.

## Abbreviations

DN: Del Nido cardioplegia
CABG: coronary artery bypass grafting
IMA: internal mammary artery
MI: myocardial infarct
LVEF: left ventricular ejection fraction
ICU: intensive care unit
ICULOS: intensive care unit length of stay
THLOS: total post-operative hospital length of stay
XC: cross lamp
CPB: cardiopulmonary bypass
MVR: Mitral Valve Replacement
AVR: aortic valve replacement
TVr: Tricuspid Valve Repair
PMI: perioperative MI
TEE: trans-esophageal echocardiographs
LV: left ventricle
RV: right ventricle
WB: whole blood
